# A New Splint for Fractured Jaw

**Published:** 1875-09

**Authors:** Francis H. Atkins


### ARTICLE VI.

### *A New Splint for Fractured Jaw.*

BY FRANCIS H. ATKINS, M. D.

On the 6th of August, 1874, I was called to see a tramp (aged about twenty-six years,) near Fort Wallace, Kansas, who, for a petty theft, had, the previous afternoon, received frontier justice at the hands of the aggrieved owner.

His head was badly bruised, cut, and swollen; the eyes closed; nose and mouth full of blood; his lower jaw fractured in two places, and the interior of the mouth so much lacerated and swollen that he could scarcely breathe or swallow. The jaw was broken (1st) at the angle on the right side, and (2d) between the left canine and left lateral incisor. The fragment—embracing the whole of the front and right side of body of jaw—was depressed, refracted, and drawn to the left side, in each direction about one-third of an inch, bringing the incisor behind the adjacent canine and below it; the right lower corner of face being strikingly depressed. At the angle there was no apparent displacement, though very free motion, crepitus, etc.

There being a complete absence of all the usual facilities for treating such cases, an attempt was made to wire the pieces together at the anterior fracture, but it failed, and on the 10th I applied a splint which I devised for the occasion, made of simple materials, that might he had at sea, etc., where such an emergency might arise.

The splint was made of a piece of hoop iron, two inches long, and from one-third to one-half inch wide, with its corners filed smooth, and four small holes punched in it to correspond respectively with the spaces between the middle incisors, and between left-middle and lateral incisors, the line of fracture, and the space between the two left bicuspids.

The object in view was to attach a brace to the left side of jaw, so that the large fragment could be drawn forward bound to it, and so be held in place. With a dental drill a small hole was made between the lateral incisors, and a space filed between the middle incisors. Between the bicuspids a natural opening existed near the gum.

The splint, being curved by stout forceps to suit the curve of the jaw, was first wired to the bicuspids through the corresponding hole in the splint; then one end of a fine, well-annealed, iron wire (suture wire) was passed into the hole in the splint corresponding to the space between the left lateral incisor and canine, and through the corresponding interdental apertures into the mouth, where the ends were seized by small forceps turned forward again and brought through the aperture between the left pair of incisors and out through the splint upon its anterior surface. By this means a loop was found for the left central and one for the left lateral incisor. As soon as gentle traction was exerted on the wire ends, the fragment moved steadily upward and forward and toward the right, with an ease that was fairly surprising when contrasted with the great digital force previously required in attempting to replace the fragment.

When the wire was drawn firmly and clinched, the replacement of the fragment was absolutely perfect, and the outline of the face fully restored, and both remained so. A Barton's occipito-parietal bandage finished the dressing. Although the patient—against orders—constantly opened his mouth for greater ease in swallowing and even masticated to a certain extent, no further adjustment was required. Owing to the splint having been a trifle wider than necessary, a small abrasion of the lip occurred after several weeks, but was easily remedied, and might have been obviated had the splint been narrower. The splint caused no discomfort, and the man often remarked that his jaw "felt so easy."

Disinfectants were used freely in the mouth until the lacerations healed. He was well in about six weeks, having worn the splint continuously. In many cases sufficient interdental spaces already exist, but the injury to the teeth in this case was trifling compared with the serious condition of the jaw. A subsequent search through all the medical literature—standard and periodical—I had access to, has made me believe this splint sufficiently novel to describe in the *Record*—that noted in the Bellevue Hospital Report, *Medical Record*, March 16, 1874, not excepted. Its simplicity would seem to recommend it in cases of much injury to the soft parts.—*Medical Record.*

